# The Ratio of Neutrophil to Lymphocyte is a Predictor in Endometrial Cancer

**DOI:** 10.1515/biol-2019-0012

**Published:** 2019-04-06

**Authors:** Yangyang Dong, Yuan Cheng, Jianliu Wang

**Affiliations:** 1 Peking University People’s Hospital, Beijing, China

**Keywords:** Neutrophil, Lymphocyte, Endometrial cancer, Prognosis

## Abstract

Objective: The aim of our study was to assess the prognostic value of the ratio of neutrophil to lymphocyte (NLR) for patients with surgically treated endometrial cancer (EC). Methods: We retrospectively reviewed 510 EC patients who were surgically treated between January 2010 and December 2016. We used receiver-operating characteristic (ROC) curve analysis to identify an optimal cut-off for NLR in predicting overall survival (OS), cancer-specific survival (CSS) and disease-free survival (DFS). Nonparametric tests were used to determine the associations between NLR and clinicopathologic characteristics. The Kaplan–Meier method and Cox proportional-hazards regression were used for survival analysis. Results: With a cut-off of 2.47, the 510 patients were divided into low NLR (NLR <2.47) and high NLR (NLR ≥2.47). Elevated NLR was associated with advanced stage (P=0.039), increased histology grade (P=0.005) and lymph node metastasis (P=0.041). Multivariable analysis suggested that NLR was an independent prognostic marker for OS (hazard ratio [HR] 4.7; 95% confidence interval [CI], 1.5-14.1; P =0.006), CSS (HR 3.6; 95% CI, 1.1-11.5; P =0.028) and DFS (HR 2.3; 95% CI, 1.0-5.2; P =0.044). Conclusion: NLR may be an independent prognostic indicator for OS, CSS and DFS. It could help clinicians with preoperative risk stratification and treatment strategy tailoring.

## 1 Introduction

Endometrial cancer (EC) is the most common gynecological malignancy in the world, accounting for 7% of all female cancers in 2016 [1]. In China, EC is the second largest gynecological tumor after cervical cancer, with 63,400 estimated new cases and 21,800 estimated deaths in 2015. The cancer incidence has increased at a rate of 3.7% per year [2]. ECs are mainly adenocarcinomas and diagnosed at an early stage. Although surgical removal and adjuvant therapy based on patient characteristic have improved the prognosis, about 20% of EC patients still show relapse and die within 5 years after surgery [3]. Also, women with advanced-stage or recurrent EC have poor clinical outcomes.

Traditionally, recognized independent prognostic factors include age, Federation of Gynecology and Obstetrics (FIGO) stage, histology grade, histopathological subtype, tumor size and lymphovascular invasion (LVSI). These factors have been widely used in risk stratification and tailoring treatment strategies and have significantly improved prognosis in EC. However, these tumor-related risk factors are not accurate enough to predict risk of EC recurrence and the outcomes. Currently, with the development of genomics, more molecular biomarkers have been found and can be used as drug targets or prognostic factors for disease recurrence or survival. Use of these biomarkers can provide novel options for patients but is time-consuming and expensive for most patients. Therefore, more effective, economical and convenient indicators are urgently needed to assist clinicians in preoperative risk stratification and administering treatment strategies.

An increasing amount of evidence supports the role of inflammation and immunology in carcinogenesis, progression and prognosis [4, 5, 6, 7, 8, 9]. Peripheral blood cells are potential biomarkers of tumor immunity and have pivotal roles in the systemic inflammatory response during all stages of malignancies.

Neutrophils represent approximately 50% to 70% of all white blood cells. They are indicators of the inflammation microenvironment state of the body, which is an important feature of various carcinomas [10, 11]. Lymphocytes are the main components of antitumor immunity. Patients with relative lymphopenia might have abnormal immune function and reduced antitumor ability, which increases the potential for cancer progression and worse outcome [12]. The ratio of neutrophil to lymphocyte (NLR), the more intuitive indicator, might be a good reflection of tumor burden and immune status.

Recently, observational studies and meta-analyses have reported elevated NLR associated with poor prognosis in various diseases, such as ovarian cancer [13], cervical cancer [14], bladder cancer [15], prostate cancer [16], colorectal cancer [17, 18], lung cancer [12, 19], pancreatic cancer [20], bipolar disorder [21] and autoimmune encephalitis [22]. However, few studies have evaluated its prognostic value in EC. Whether NLR is associated with outcomes for EC is unclear. Here, we investigated the prognostic significance of NLR in patients with surgically treated EC.

## 2 Materials and methods

### 2.1 Patients

Retrospective data were collected for patients who underwent surgical staging procedures at Peking University People’s Hospital between January 2010 and December 2016. Patients with inflammatory disease, hematological disease, autoimmune disease, or concurrent second malignancies or who were missing preoperative complete blood cell counts or complete blood cell counts performed more than 1 week before surgery were excluded.

The surgical procedures consisted of at least total hysterectomy (TAH) and bilateral salpingo-oophorectomy (BSO). TAH, BSO and systematic lymphadenectomy were performed for patients with FIGO stage IB or higher, grade 3 endometrioid adenocarcinoma, non-endometrioid histology, LVSI or tumor > 2 cm. Postoperative adjuvant treatments were tailored to the pathology findings in accordance with the institutional treatment guidelines and FIGO guidelines. Adjuvant chemotherapy or radiotherapy was recommended for patients with any recurrence risk factors. The cancer was staged according to the 2009 FIGO guidelines and was graded according to the FIGO classification. Data on age, complete blood cell count within 1 week before surgery, FIGO stage, histologic grade, histopathological subtype, presence of LVSI, lymph node status, peritoneal cytology, history of adjuvant chemotherapy and radiotherapy were obtained from medical records.

The NLR was defined as absolute neutrophil count divided by absolute lymphocyte count. Overall survival (OS) was defined as the time between the date of hysterectomy-based surgical staging and the date of death or the last follow-up if the patient was alive. Cancer-specific survival (CSS) was defined as the time between the date of hysterectomy-based surgical staging and the date of death due to EC or the last follow-up if the patient was alive. Disease-free survival (DFS) was defined as the time between hysterectomy-based surgical staging and the date of first recurrence or last follow-up if there was no recurrence [23]. The primary endpoint was OS. Secondary endpoints included CSS and DFS.

**Informed consent**: Informed consent has been obtained from all individuals included in this study.

**Ethical approval**: The research related to human use has been complied with all the relevant national regulations, institutional policies and in accordance the tenets of the Helsinki Declaration, and has been approved by the ethics committee of Peking University People’s Hospital (approval no. 2016PHB054-01).

### 2.2 Statistical analysis

Categorical data were compared by chi-square test or two-tailed Fisher exact test, as appropriate. The optimal cut-off value was estimated by using the package Cut-off Finder in R software. We plotted receiver operating characteristic (ROC) curves of NLR for OS, CSS and DFS. An optimal cut-off value that maximized the sum of sensitivity and specificity in the ROC curve was used. Survival was analyzed by the Kaplan–Meier method, and significant differences in survival were identified by log-rank test. Univariable and multivariable analyses involved Cox proportional-hazards models, estimating hazard ratios (HRs) and 95% confidence intervals (CIs). All statistical tests involved use of R v2.12.2. All statistical analyses were two-tailed, and p<0.05 was considered statistically significant.

## 3 Results

### 3.1 Patient populations and clinicopathologic characteristics

The clinicopathologic characteristics of the 510 patients are summarized in Table 1. The median age at diagnosis was 56 years (range 23–83). All patients underwent hysterectomy-based surgical staging for EC. In total, 406 (79.6%), 25 (4.9%), 64 (12.6%), and 15 (2.9%) patients had stage I, II, III, and IV disease, respectively. Most ECs had endometrioid histology (86.3%) and were low grade (grade 1–2, 76.7%). Overall, 81% of the patients underwent lymphadenectomy (pelvic/para-aortic) and 26.5% received adjuvant chemotherapy, 2.4% adjuvant radiotherapy and 14.9% simultaneous adjuvant chemotherapy and radiotherapy. In total, 48 (11.6%) patients had lymph node involvement and 96 (18.8%) had LVSI. The median NLR was 2.30 (range 0.70–13.04) and median follow-up was 41 months (range 1–91 months). In all, 22 patients died from cancer-related causes, and 3 from suicide; 39 had tumor recurrence.

**Table 1:** Clinicopathologic characteristics of patients with endometrial cancer (n=510).

Characteristic		n	(%)

Age (year) median (range)		56	23-83
FIGO stage			
	I	406	79.6%
	II	25	4.9%
	III	64	12.6%
	IV	15	2.9%
Histology grade			
	G1	171	33.5%
	G2	220	43.1%
	G3	119	23.3%
Histology type			
	Endometrioid	440	86.3%
	Nonendometrioid	70	13.7%
LN metastasis			
	Absent	365	88.4%
	Present	48	11.6%
Peritoneal cytology			
	Absent	352	91.0%
	Present	35	9.0%
LVSI			
	Absent	414	81.2%
	Present	96	18.8%
NLR median (range)		2.3	0.70-13.04

FIGO=International Federation of Gynaecology and Obstetrics; LN=lymph node; LVSI=lymphovascular space invasion; NLR=neutrophil:lymphocyte ratio

### 3.2 Association between NLR and clinicopathologic factors

Cut-off values of NLR for prognostication in EC range from 2.4 to 4.68 [24, 25]. At present, there is no definitive cut-off value. Therefore, we used an optimal cut-off value that maximized the sum of sensitivity and specificity in the ROC curve. For OS, the best NLR cut-off was 2.47, with 76.0% sensitivity and 56.3% specificity. The area under the ROC curve (AUC) was 0.68. For CSS, the best cut-off was 2.62, with 68.2% sensitivity and 61.3% specificity. The AUC was 0.66. For DFS, the best cut-off was 2.47, with 66.7% sensitivity and 56.1% specificity. The AUC was 0.60. The optimal cutoff for OS and DFS were both 2.47. When using 2.47 as the cut-off for CSS, the AUC was 0.66. The cut-offs 2.47 and 2.62 for CSS had the almost same AUC value. So, we chose 2.47 as the cut-off for OS, CSS and DFS.

The NLR cut-off was used to divide the patients into high NLR (NLR ≥2.47) and low NLR (NLR <2.47). The associations between clinicopathologic factors and NLR are in Table 2. With patients sorted into binary sets (high/low, positive/negative) for various clinicopathologic factors, NLR was significantly associated with FIGO stage (P<0.039), histology grade (P*=*0.005), and lymph node metastasis (P*=*0.041).

**Table 2:** Clinical and pathological characteristics of patients with endometrial cancer by high and low NLR (n=510).

Characteristic	NLR-low(<2.47)		NLR-high(≥2.47)		P-value
	n	(%)	n	(%)	
Age(year)					0.768
<60	184	65.7%	154	67.0%	
≥60	96	34.3%	76	33.0%	
FIGO stage					**0.039**
I–II	245	87.5%	186	80.9%	
III–IV	35	12.5%	44	19.1%	
Histology grade					**0.005**
G1/G2	228	81.4%	163	70.9%	
G3	52	18.6%	67	29.1%	
Histology type					0.055
Endometrioid	249	88.9%	191	83.0%	
Nonendometrioid	31	11.1%	39	17.0%	
LN metastasis					**0.041**
Absent	209	91.3%	156	84.8%	
Present	20	8.7%	28	15.2%	
Peritoneal cytology					0.070
Absent	197	93.4%	155	88.1%	
Present	14	6.6%	21	11.9%	
LVSI					0.194
Absent	233	83.2%	181	78.7%	
Present	47	16.8%	49	21.3%	

NLR=neutrophil:lymphocyte ratio; FIGO=International Federation of Gynaecology and Obstetrics; LN=lymph node; LVSI= lymphovascular space invasion. Significant p values are in bold, by chi-square test

### 3.3 Univariable and multivariable analyses for OS, CSS and DFS (Tables 3 to 5, Figure 1)

**Figure 1:**
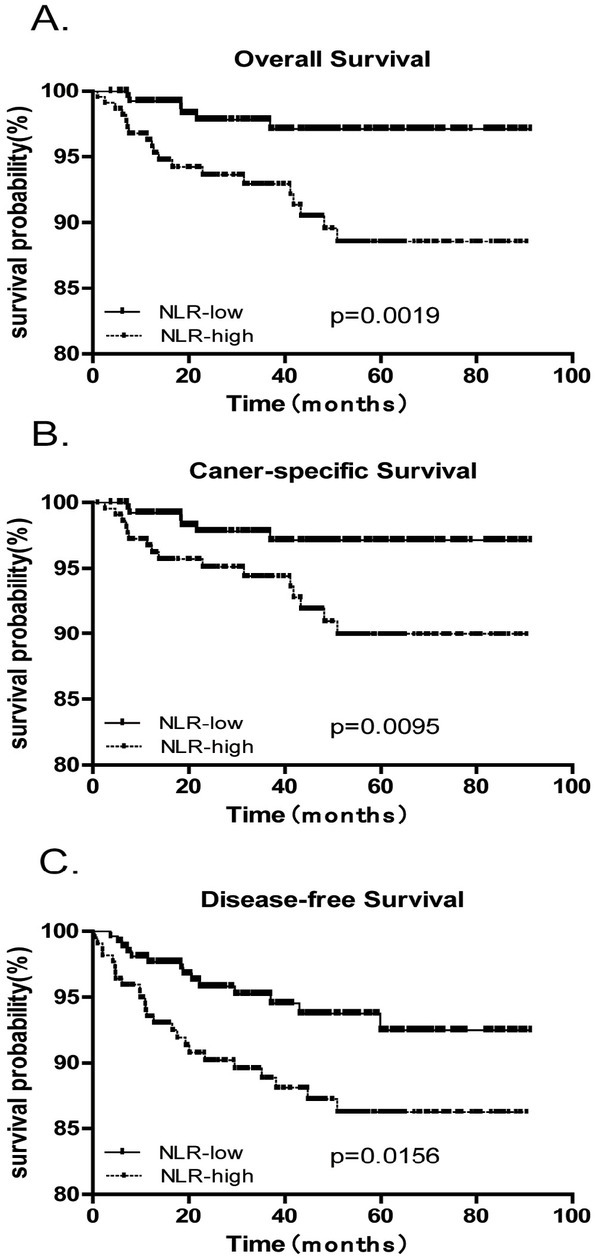
Kaplan–Meier curves for OS, CSS and PFS with endometrial cancer. (A) Kaplan–Meier curves for overall survival (OS). (B) Kaplan–Meier curves for cancer-specific survival (CSS). (C) Kaplan–Meier curves for disease-free survival (DFS).

**Table 3:** Univariable and multivariable analysis of factors, including NLR cut-off, associated with overall survival with endometrial cancer.

Characteristic		Univariable		Multivariable	
		HR (95% CI)	P-value	HR (95% CI)	P-value
Age(year)					
	<60	1 (Reference)		1 (Reference)	
	≥60	3.1 (1.4–6.9)	**0.006**	4.7 (1.7–13.1)	**0.003**
FIGO stage					
	I–II	1 (Reference)		1 (Reference)	
	III–IV	21.8 (8.7–54.7)	**<0.001**	5.4 (1.8–15.7)	**0.002**
Histology grade					
	G1/G2	1 (Reference)		1 (Reference)	
	G3	15.4 (5.8–41.0)	**<0.001**	2.6 (0.6–11.1)	0.207
Type					
	Type I	1 (Reference)		1 (Reference)	
	Type II	11.6 (5.2–25.8)	**<0.001**	1.2 (0.3–5.0)	0.763
Peritoneal cytology					
	Absent	1 (Reference)		1 (Reference)	
	Present	10.5 (4.4–25.2)	**<0.001**	2.7 (0.9–8.3)	0.084
LVSI					
	Absent	1 (Reference)		1 (Reference)	
	Present	8.2 (3.7–18.4)	**<0.001**	2.6 (0.9–7.3)	0.071
NLR					
	Low	1 (Reference)		1 (Reference)	
	High	3.9 (1.5–9.7)	**0.004**	4.7 (1.5–14.1)	**0.006**

NLR=neutrophil:lymphocyte ratio; HR=hazard ratio; 95% CI=95% confidence interval; FIGO=International Federation of Gynaecology and Obstetrics; LN=lymph node; LVSI= lymphovascular space invasion. Significant p values are in bold, by chi-square test; Bold values indicate statistical significance.

Prognostic variables for univariable analysis included age, FIGO stage, histology grade, histopathological subtype, peritoneal cytology and presence of lymphovascular space invasion, a known independent prognostic indicator for EC. Depth of myometrial invasion, cervical involvement and lymph-node status form part of the FIGO staging system and, as such, were not included as independent variables in the analysis.

Kaplan–Meier analysis for OS, CSS and DFS (Figure 1) revealed significantly worse OS, CSS and DFS for patients with high preoperative NLR. On univariable analysis, factors associated with OS, CSS and DFS were age (P=0.006, P=0.011 and P=0.033), FIGO stage (all P<0.001), histology grade (all P<0.001), histopathology subtype (all P<0.001), peritoneal cytology (all P<0.001), LVSI (all P<0.001), and NLR (P=0.014, P=0.004 and P=0.018). On multivariable analysis, factors associated with OS were age (HR 4.7; 95% CI, 1.7-13.1; P=0.003), FIGO stage (HR 5.4; 95% CI, 1.8-15.7; P=0.002), and NLR (HR 4.7; 95% CI, 1.5-14.1; P=0.006) (Table 3). Independent predictors of CSS were also age, FIGO stage and NLR [HR 4.5 (95% CI, 1.5-13.6; P =0.008), HR 5.6 (95% CI, 1.7-18.3; P =0.004) and HR 3.6 (95% CI, 1.1-11.5; P =0.028), respectively] (Table 4). Independent predictors of DFS were age (HR 2.2; 95% CI, 1.0-4.8;P = 0.045), FIGO stage (HR 4.4; 95% CI, 1.8-10.5; P =0.001), histology grade (HR 4.2; 95% CI, 1.3-13.5;P = 0.016), peritoneal cytology (HR 3.2; 95% CI, 1.2-8.2; P =0.016) and NLR (HR 2.3; 95% CI, 1.0-5.2; P =0.044) (Table 5).

**Table 4:** Univariable and multivariable analysis of factors, including NLR cut-off, associated with cancer-specific survival with endometrial cancer.

Characteristic		Univariate		Multivariate	
		HR (95% CI)	P-value	HR (95% CI)	P-value
Age(year)					
	<60	1 (Reference)		1 (Reference)	
	≥60	3.0 (1.3- 7.0)	**0.011**	4.5 (1.5- 13.6)	**0.008**
FIGO stage					
	I–II	1 (Reference)		1 (Reference)	
	III–IV	23.8 (8.8- 64.5)	**<0.001**	5.6 (1.7- 18.3)	**0.004**
Histology grade					
	G1/G2	1 (Reference)		1 (Reference)	
	G3	17.4 (5.9- 51.5)	**<0.001**	2.8 (0.5- 15.6)	0.236
Histology type					
	Endometrioid	1 (Reference)		1 (Reference)	
	Nonendometrioid	13.5 (5.7- 32.3)	**<0.001**	1.5 (0.3- 7.9)	0.618
Peritoneal cytology					
	Absent	1 (Reference)		1 (Reference)	
	Present	11.5 (4.5- 29.5)	**<0.001**	2.9 (0.9- 9.5)	0.088
LVSI					
	Absent	1 (Reference)		1 (Reference)	
	Present	6.7 (2.9- 15.6)	**<0.001**	1.7 (0.6- 5.4)	0.350
NLR					
	Low	1 (Reference)		1 (Reference)	
	High	3.2 (1.3- 8.3)	**0.014**	3.6 (1.1- 11.5)	**0.028**

NLR=neutrophil:lymphocyte ratio; HR=hazard ratio; 95% CI=95% confidence interval; FIGO=International Federation of Gynaecology and Obstetrics; LN=lymph node; LVSI= lymphovascular space invasion. Significant p values are in bold, by chi-square test; Bold values indicate statistical significance.

**Table 5:** Univariable and multivariable analysis of factors, including NLR cut-off, associated with disease-free survival with endometrial cancer.

Characteristic		Univariate		Multivariate	
		HR (95% CI)	P-value	HR (95% CI)	P-value
Age(year)					
	<60	1 (Reference)		1 (Reference)	
	≥60	2.0 (1.1- 3.7)	**0.033**	2.2 (1.0- 4.8)	**0.045**
FIGO stage					
	I–II	1 (Reference)		1 (Reference)	
	III–IV	11.6 (6.1- 22.2)	**<0.001**	4.4 (1.8- 10.5)	**0.001**
Histology grade					
	G1/G2	1 (Reference)		1 (Reference)	
	G3	11.6 (5.6- 23.7)	**<0.001**	4.2 (1.3- 13.5)	**0.016**
Histology type					
	Endometrioid	1 (Reference)		1 (Reference)	
	Nonendometrioid	9.3 (4.9- 17.4)	**<0.001**	1.0 (0.3- 3.2)	0.961
Peritoneal cytology					
	Absent	1 (Reference)		1 (Reference)	
	Present	9.9 (4.7- 20.7)	**<0.001**	3.2 (1.2- 8.2)	**0.016**
LVSI					
	Absent	1 (Reference)		1 (Reference)	
	Present	5.3 (2.8- 10.0)	**<0.001**	1.1 (0.4- 2.6)	0.908
NLR					
	Low	1 (Reference)		1 (Reference)	
	High	2.2 (1.1- 4.2)	**0.018**	2.3 (1.0- 5.2)	**0.044**

NLR=neutrophil:lymphocyte ratio; HR=hazard ratio; 95% CI=95% confidence interval; FIGO=International Federation of Gynaecology and Obstetrics; LN=lymph node; LVSI= lymphovascular space invasion. Significant p values are in bold, by chi-square test; Bold values indicate statistical significance.

## 4 Discussion

Neutrophils are indicators of the whole inflammation microenvironment state of the body, and lymphocytes are the main components of antitumor immunity. Lymphocytes are generally reduced in various types of tumors [26, 27], Patients with relative lymphopenia might have abnormal immune function and reduced antitumor ability, which increases the potential of cancer progression and worse outcome [12]. As in our study, the neutrophil count in the death group was higher than that in the survival group (4.5*109/L VS 4.3*10^9^ /L), and the lymphocyte count in the death group was lower than in that in the survival group (1.8* 109/L VS 1.4*109/L). The ratio of neutrophil to lymphocyte (NLR), the more intuitive indicator, might be a good reflection of tumor burden and immune status.

The NLR is an important feature of the systemic inflammatory response [28]. Elevated NLR has been associated with poor prognosis in many solid tumors [29]. Increased NLR during treatment was found to be associated with low response rate and poor outcomes in metastatic renal cell carcinoma [30]. Here we found elevated NLR as an independent prognostic factor for OS as well as CSS and DFS. Recently, Haruma et al. and Cummings et al. found elevated NLR as a prognostic factor for poor OS and CSS [24, 31], which is in agreement with our study; however, Takahashi et al. found leukocytosis (neutrophil count ≥7200/μl) but not NLR as an independent predictor of survival outcome [32]. In the Li et al. study, NLR was associated with poor OS in the log-rank test but was not significant on multivariable analysis [25]. The selection of the cut-off value for NLR might explain the different findings.

Even though elevated NLR was found to be associated with poor prognosis in many cancers, the best cut-off value is still unknown. The cut-off values were mostly determined by the ROC curve and had a wide range (1.95.0) in different cancers [33]. The cut-off ranged from 2.1 to 3.0 in bladder cancer [15, 34, 35], 2.0 to 5.0 in colorectal cancer [18, 36, 37], 2.5 to 5.0 in lung cancer [19, 38, 39], 1.9 to 5.0 in cervical cancer [40, 41, 42], and 2.4 to 4.68 in EC [24, 25]. We used an optimal cut-off that maximized the sum of sensitivity and specificity in the ROC curve in our study. For OS, CSS and DFS, the best cut-off for NLR was 2.47, 2.62, and 2.47, respectively, and the corresponding AUC was 0.68, 0.66, and 0.60, respectively. In predicting CSS, the cut-offs 2.47 and 2.62 had almost the same AUC value. We chose a concordant and optimization process for the NLR cut-off of 2.47 to predict OS, CSS and DFS in EC. Cummings et al. chose 2.4 for the NLR cut-off according to ROC curves for OS and CSS, and Haruma et al. chose 2.41 and 2.70 as cut-off values for DFS and OS according to ROC curves, respectively. Both studies demonstrated the association between elevated NLR and survival outcomes in EC. Takahashi et al. chose a mean value (3.0) as a cut-off, and Li et al. chose 4.68 (cited from other cancer

studies); neither found an association between NLR and survival outcomes. Our study found NLR (cut-off 2.47) as an independent prognostic factor for OS and also for CSS and DFS in this cohort of EC patients.

Previous studies have shown a strong association between elevated NLR and poor prognosis in various cancers. We found that elevated NLR is significantly

associated with advanced FIGO stage, increased histology grade, and lymph node metastasis, which can worsen the prognosis. The mechanism underlying neutrophilia and lymphopenia and carcinogenesis as well as tumor progression is still unclear. Neutrophils can promote tumorogenesis and cancer development by multiple mechanisms. They can increase DNA mutations via the release of reactive oxygen species; facilitate cancer cell proliferation via the secretion of cytokines and chemokines; promote vascularization via the secretion of vascular endothelial growth factor; enhance tumor cell invasiveness and metastasis by secreting proteases, elastase and matrix metallopeptidase 9 (MMP-9); and suppress effective antitumor immunity of CD8+ T cells via the release of nitric oxide synthase and arginase [29]. Lymphocytes have potent anti-cancer activity, and they play an important role in the immune defense against tumor cells [43]. Previous studies have found an association of decreased tumor-infiltrating lymphocyte count with worse survival outcomes in colorectal cancer [44], esophageal cancer [45] and ovarian cancer [46].

Elevated NLR is due to relative increase in neutrophil count or relative decrease in lymphocyte count. The imbalance of neutrophils and lymphocytes in the tumor microenvironment plays a role in cancer progression. NRL might be a circulatory marker reflecting increased cancer aggressiveness.

Our results suggest that survival of patients with EC depends on traditional risk factors but is also affected by pre-treatment NLR, so inhibiting the inflammatory reaction and improving the immune ability could improve prognosis with EC. Moreover, NLR is calculated by use of an inexpensive, routine and convenient test, and it can provide useful information for management and treatment outcomes with EC. Our data revealed that NLR (cut-off 2.47) may be an independent prognostic factor for OS and also for CSS and DFS.

However, our study has some limitations. First, although 510 patients were enrolled and the data were from 2010-2016, the median follow-up duration (median 41 months, range 1–91) was rather short. As well, the retrospective clinical data with uncertain potential confounding factors may have negatively affected the accuracy of the results but is inevitable. Large prospective multi-institutional studies with longer follow-up periods are needed to clarify the significance of our findings.

In conclusion, our study suggests the potential value of pre-treatment NLR as a predictor of prognosis with EC. The calculation of NLR is inexpensive, routine and convenient and could help clinicians with preoperative risk stratification and treatment strategy administration.
